# Retargeted human avidin-CAR T cells for adoptive immunotherapy of EGFRvIII expressing gliomas and their evaluation via optical imaging

**DOI:** 10.18632/oncotarget.4362

**Published:** 2015-06-08

**Authors:** Kaiyu Liu, Xujie Liu, Zhiping Peng, Haojie Sun, Mingzhi Zhang, Jianning Zhang, Shuang Liu, Limin Hao, Guoqiu Lu, Kangcheng Zheng, Xikui Gong, Di Wu, Fan Wang, Li Shen

**Affiliations:** ^1^ Department of Biochemistry and Molecular Biology, Peking University Health Science Center, Beijing, People's Republic of China; ^2^ Department of Radiological Medicine, Chongqing Medical University, Chongqing, People's Republic of China; ^3^ Department of Cell Biology, Peking University Health Science Center, Beijing, People's Republic of China; ^4^ Peking University Stem Cell Research Center, Beijing, People's Republic of China; ^5^ Department of Neurosurgery, The Chinese PLA Navy General Hospital, Beijing, People's Republic of China; ^6^ Beijing Cellonis Biotechnologies Co., Ltd, Zhongguancun Bio-Medicine Park, Beijing, People's Republic of China; ^7^ Medical Isotopes Research Center, Peking University, Beijing, People's Republic of China; ^8^ Department of Radiation Medicine, School of Basic Medical Sciences, Peking University, Beijing, People's Republic of China

**Keywords:** adoptive immunotherapy, avidin-CAR, biotinylated, EGFRvIII, optical imaging

## Abstract

There has been significant progress in the design of chimeric antigen receptors (CAR) for adoptive immunotherapy targeting tumor-associated antigens. However, the challenge of monitoring the therapy in real time has been continually ignored. To address this issue, we developed optical molecular imaging approaches to evaluate a recently reported novel CAR strategy for adoptive immunotherapy against glioma xenografts expressing EGFRvIII. We initially biotinylated a novel anti-EGFRvIII monoclonal antibody (biotin-4G1) to pre-target EGFRvIII^+^ gliomas and then redirect activated avidin-CAR expressing T cells against the pre-targeted biotin-4G1. By optical imaging study and bio-distribution analysis, we confirmed the specificity of pre-target and target and determined the optimal time for T cells adoptive transfer *in vivo*. The results showed this therapeutic strategy offered efficient therapy effect to EGFRvIII^+^ glioma-bearing mice and implied that optical imaging is a highly useful tool in aiding in the instruction of clinical CAR-T cells adoptive transfer in future.

## INTRODUCTION

The epidermal growth factor receptor variant III (EGFRvIII) is an oncogenic variant of EGFR that is exclusively expressed by malignant tumors. EGFRvIII causes constitutive phosphorylation of tyrosine kinases and enhances the malignancy of gliomas [1-5]. The expression of EGFRvIII within a cell is associated with survival, invasion, angiogenesis and resistance against radiation and chemotherapy [6-9]. EGFRvIII is, therefore, an attractive target for cancer immunotherapy [10-13].

Chimeric antigen receptor (CAR)-based adoptive immunotherapy employs T lymphocytes that are genetically modified to express CARs that combine both specific targeting of antibodies and T-cells mediated immune responses, which enable T cells to target cell surface antigens independent of MHC restriction [14-16]. Typically, CARs are designed to consist of a single chain antibody fragment (scFv) that are fused to extracellular spacer and transmembrane domains, which can contain various combinations of cytoplasmic signaling moieties, such as CD3ζ, CD28, OX40 or 4-1BB [16-19]. The scFv are usually derived from monoclonal antibodies that are prepared to specifically direct against tumor-associated antigens (TAAs) expression on cancer cells membrane. Recently, a study reported a novel and universal CAR strategy that extends the recognition specificity potential of CAR-T cells by using a biotin-avidin system [20, 21]: First, the TAAs are pre-targeted with biotinylated molecules, such as monoclonal antibodies, peptides, ligands or enzyme substrates. Then, T lymphocytes expressing a CAR that contains avidin (avidin-CAR) instead of a scFv linking the intracellular T cell signaling domain are then adoptively transferred. In this study, we used this novel strategy to treat EGFRvIII expressing gliomas and evaluate the therapies progress through bioluminescent imaging: We first biotinylated a novel anti-EGFRvIII monoclonal antibody, 4G1 (biotin-4G1), and bioengineered the expression of avidin-CAR on T cells. Next, we pre-targeted xenograft EGFRvIII expressing gliomas with biotin-4G1 and then targeted biotin-4G1 with avidin-CAR-T cells. More importantly, in this study we firstly employed optical molecular imaging approach to evaluate the specificity of pre-target or target and determine the right time for T cells transfer, as well. We labeled biotin-4G1 with near-infrared dye to get biotin-4G1-dye and then injected it into EGFRvIII^+^ or EGFRvIII^−^ gliomas to confirm the accumulation of biotin-4G1 in tumors and normal organs. The results indicated that our strategy offered a specific and efficient cytotoxicity against an EGFRvIII expressing gliomas, both *in vitro* and *in vivo*, and optical molecular imaging can facilitate the observation and evaluation of the therapy.

## RESULTS

### Avidin-CAR was constructed and expressed on activated T cells

**Figure 1:**
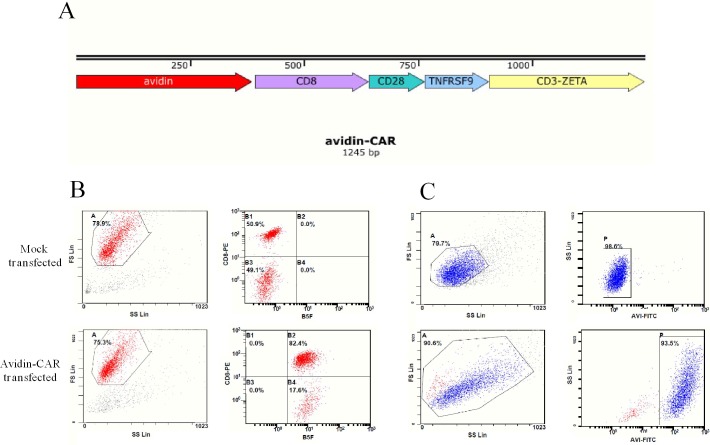
Plasmids construction and avidin expression on T cells **A.** Schematic representations of pITA-Avadin-CAR. Avidin expression efficiency of mock or avidin-CAR infection was detected by flow cytometry with B5F **B.** or mouse anti-avidin IgG **C.**

Lentiviral plasmid pITA-Avadin-CAR was constructed as shown in Figure 1A. Avidin-CAR encodes a fusion protein consisting of streptavidin, the hinge and transmembrane region of human CD8, the costimulatory signal transduction domain of CD28 and 4-1BB, and the intracellular signal domain of CD3ζ. After 12 d of lentiviral infection, avidin-CAR expression efficiency reached 100% and 93.5% as determined by B5F and anti-avidin antibody analysis, respectively (Figure 1B).

### Phenotypic and cytokine analysis

**Figure 2:**
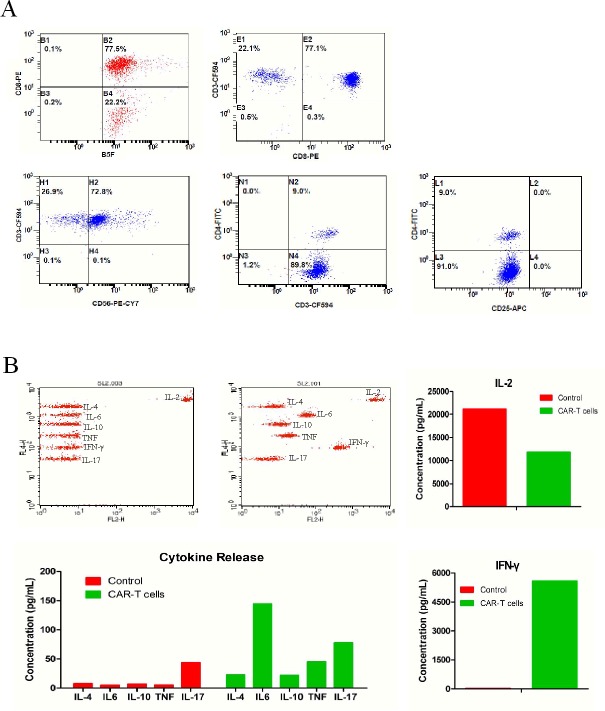
Phenotypes and cytokine release of avidin-CAR T cells **A.** Expression of phenotypic markers including CD8, CD3, CD4, CD56, CD25 on avidin-CAR-T were determined by combinational flow cytometric analysis. **B.** Concentration of cytokines released by avidin-CAR T cells was determined by cytometric bead array (top middle panel); Cytokines concentration of non-activated avidin-CAR-T cells was also determined as control (top left panel).

To determine the characteristics of T cells expressing avidin-CAR for adoptive transfer, immune-phenotypes of cells were determined after 12d of infection. Classic cytokine-induced killer (CIK) phenotypes were confirmed again in this study, which showed that 72.8% of the T cells expressed CD3^+^CD56^+^ [23] (Figure 2A). Furthermore, results showed that 77.1% of the population were CD8^+^CD3^+^ cells (cytotoxic T cells), whereas, CD3^+^CD4^+^ (helper T cells) and CD4^+^CD25^+^ [24] (regulatory T cells or activated CD4 T cells) cells only accounted for 9 and 0% of the population, respectively. This suggests that the cells would favor a cytotoxic function.

Cytokine release by avidin-CAR-T cells from No.1 samples was analyzed by Cytometric Bead Array. In comparison to the control, increased IFN-γ (2,961.1 vs. 24.4 pg/ml), IL-4 (45.3 vs. 7.7 pg/ml), IL-6 (48.5 vs. 4.9 pg/ml), IL-10 (21.1 vs. 6.8 pg/ml), TNF-Ρ (33.7 vs. 5.5 pg/ml) and IL-17 (162.1 vs. 43.4 pg/ml) were produced by the avidin-CAR-T cells, suggesting their activation was effective. Lower amounts of IL-2 (15,868.7 vs 21,120.4 pg/ml) were detected in the avidin-CAR-T cell culture, which may be the result IL-2 uptake by the activated T cells (Figure 2B). More sample testing results were summarized in Supplemental Table 1.

### *In vitro* determination of pre-target and re-target

Through labeling preparation and calculation, the moles biotin conjugated to per mole 4G1 are approximately equal to 7.2; and the moles dye conjugated to per mole biotin-4G1 are approximately equal to 3.5.

**Figure 3:**
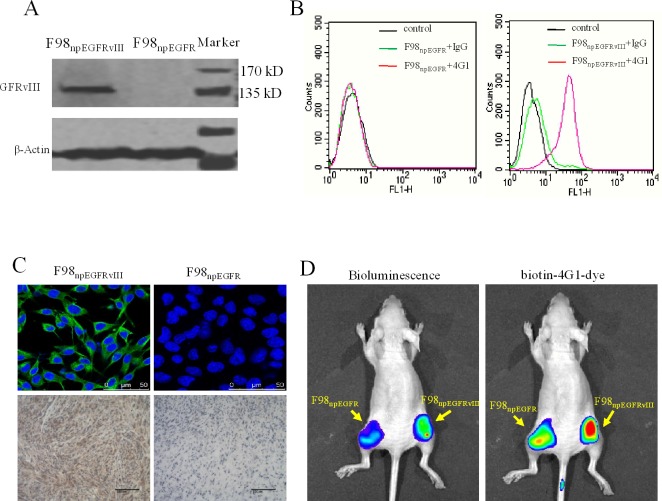
Biotinylated 4G1 exclusively recognizes with EGFRvIII **A.** western-blotting, **B.** flow cytometry, **C.** IFA and IHC were undertaken for EGFRvIII detection. Biotin-4G1 was used as primary antibody. **D.** Biotin-4G1-dye as an optical molecular probe was intravenous injected and specifically uptaken by EGFRvIII+ xenograft tumor. Left panel: bioluminescent imaging after luciferin intraperitoneal injection; right panel: optical imaging at Ex/Em: 675/720 nm for biotin-4G1-dye detection.

**Figure 4:**
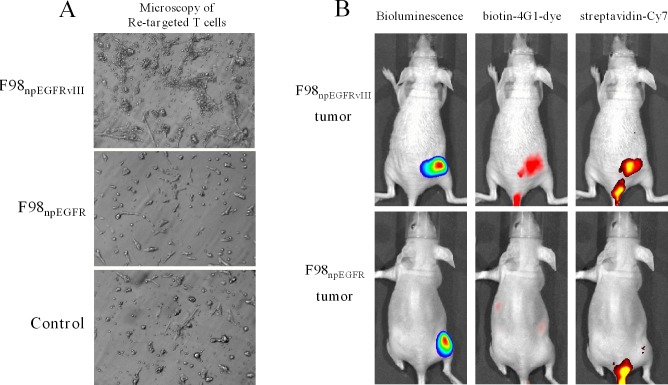
Avidin-CAR T cells re-target biotin-4G1 **A.** Microscopy observation of avidin-CAR T cells' re-targeted to F98_npEGFRvIII_ (upper) or F98_npEGFR_ (middle) cells pre-targeted with biotin-4G1. F98_npEGFRvIII_ pre-targeted with 4G1 was served as control (lower). **B.** Optical molecular imaging for pre-target and re-target evaluation. Left panel: bioluminescent imaging after luciferin intraperitoneal injection; middle panel: optical imaging at Ex/Em: 675/720 nm for biotin-4G1-dye detection; right panel: optical imaging at Ex/Em: 750/785 nm for Streptavidin-Cy7 detection.

Results of western blotting (Figure 3A), flow cytometric analysis (Figure 3B), IFA and IHC (Figure 3C) determined that biotin-4G1 exclusively bound to EGFRvIII expressed by F98_npEGFRvIII_ cells but not wild-type EGFR expressed by F98_npEGFR_ cells (Figure 3A–3C). *In vitro* cell binding results showed that avidin-CAR-T cells targeted F98_npEGFRvIII_ cells that were bound with biotin-4G1, whereas, few avidin-CAR-T cells could be observed on F98_npEGFR_ cells pre-targeted with biotin-4G1 (Figure 4A).

### Optical imaging evaluation of pre-target and re-target

As shown in Figure 3D, biotin-4G1-dye did not effectively bind to F98_npEGFR_ tumor, confirming that biotin-4G1 specifically pre-targets to EGFRvIII^+^ tumor in an antigen-dependent manner *in vivo*. Additionally, the optical imaging of streptavidin-Cy7 uptake in EGFRvIII tumors demonstrated the stability of biotin-4G1 and indicated that avidin-CAR-T cells can efficiently target EGFRvIII^+^ tumors pre-targeted with biotin-4G1 (Figure 4B).

### Optimal time point for avidin-CAR-T cell transfer

**Figure 5:**
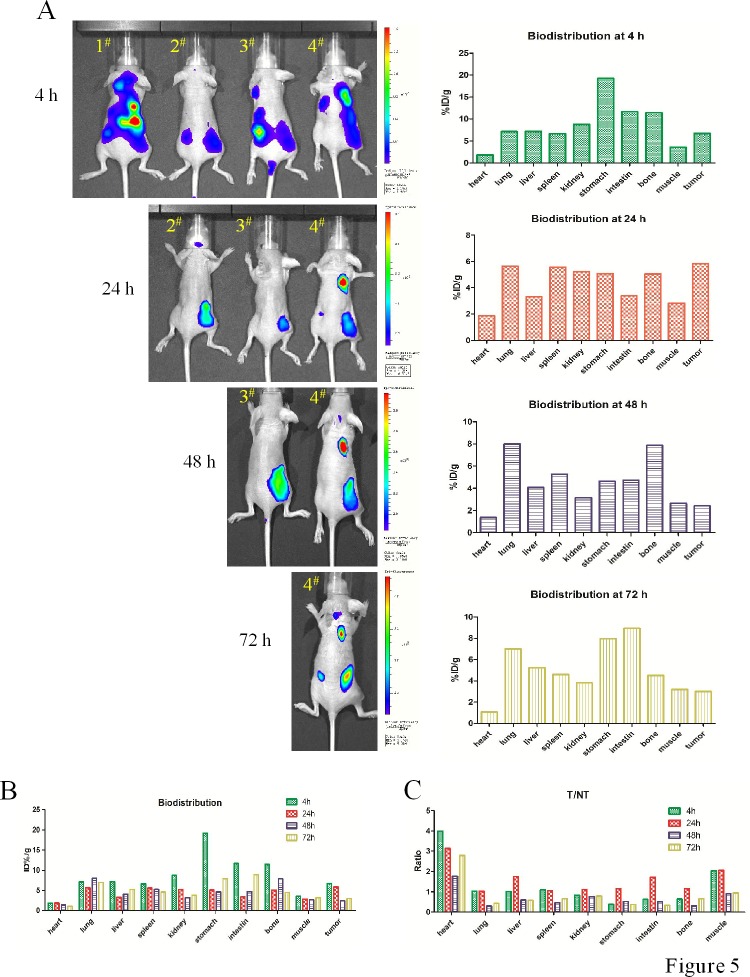
Optical imaging for determining time point of CAR-T cells transfer **A.** Left panel: 4 mice injected with biotin-4G1-dye were undertaken optical imaging study at 4, 24, 48 and 72 h post-injection. Right panel: the corresponding bio-distribution analysis at those time point. **B.** Summarized bio-distribution analysis for all time points. **C.** The ratio of %ID/g between tumor and normal tissue (T/NT) analysis.

Representative successive optical images of EGFRvIII^+^ tumor-bearing mice at different times after intravenous injection of 0.5 nmol biotin-4G1-dye are shown in Figure 5A. From the imaging results, all four tumors can't be well visualized at 4 h after biotin-4G1-dye injection mainly due to prominent uptake of tracer by some normal organs. Start from 24 h, tumors can be clearly visible with good tumor-to-background contrast.

Quantification of tracer accumulation in tumors and major organs was realized by bio-distribution test. As shown in Figure 5B and Supplemental Table 3, the tumor uptakes were 6.68, 5.85, 2.41 and 2.99 %ID/g at 4, 24, 48 and 72 h post-injection, respectively (the weight and radiant efficiency of tumors and major organs were summarized in Supplemental Table 2 and Figure 1). Higher accumulation of biotin-4G1-dye in tumors at 4 and 24 h compared to other time points implies more avidin-CAR-T cells reach tumor at early time. However, accumulation of tracer in normal tissues is almost higher at 4 h than that of 24 h, therefore as shown in Figure 5C and Supplemental Table 4, the values of T/NT at 24 h are relatively higher, suggesting 24 h is supposed to be the optimal time point for avidin-CAR-T cell transfer both because of higher tracer accumulation in tumor and relative lower normal tissues uptake.

### Antitumor efficacy evaluation

**Figure 6:**
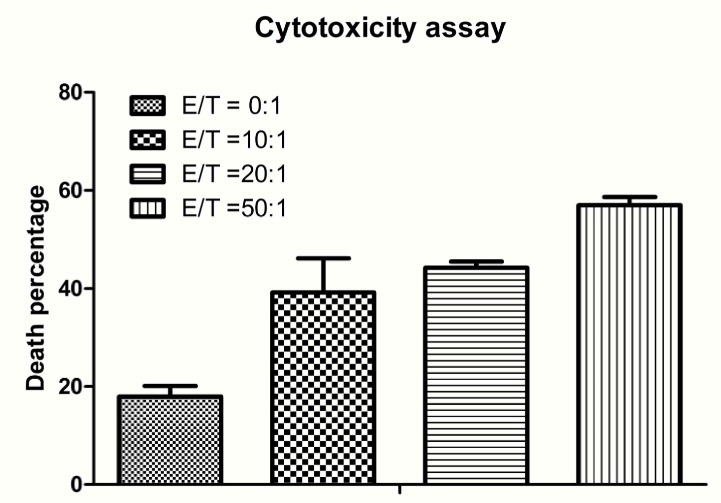
Cytotoxicity assay for therapy efficacy determination Increasing numbers of avidin-CAR T cells (effector cells) were added to pre-targeted cells at effector-to-target ratios (E:T) of 0:1, 10:1, 20:1 and 50:1. The percentage of PI stained cells was analyzed by flow cytometric analysis. The p values of E:T at 0:1 and 20:1, 0:1 and 50:1, 20:1 and 50:1 were both lower than 0.001 (_***_); *p* values of 0:1 and 10:1, 10:1 and 50:1 were 0.072 (_**_) and 0.0127 (_*_), respectively; and p values of 10:1 and 20:1 was 0.2834 (no significant difference).

To determine whether avidin-CAR-T cells are capable of imposing cytotoxicity against EGFRvIII positive cells, effector cells (avidin-CAR-T) were cultured with target cells (F98_npEGFRvIII_ cells). The cytotoxicity assay results showed that the effector cells exerted significant cytotoxicity against F98_npEGFRvIII_ cells, depending on the E:T ratio. The percentage of target cells (F98_npEGFRvIII_ cells) that were killed increased with an increase in the E:T ratio and reached almost 60% when the E:T ratio was 50:1 (Figure 6).

**Figure 7:**
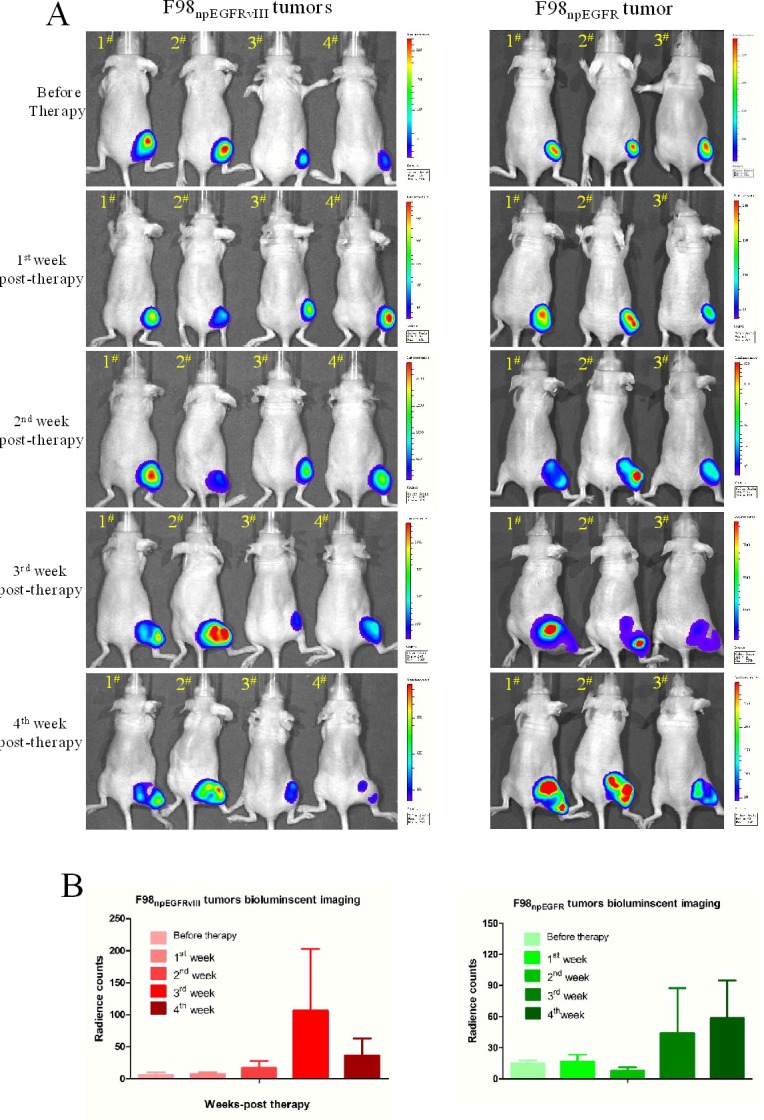
Bioluminescent imaging for therapy efficacy determination **A.** Successive bioluminescent imaging to monitor avidin-CAR-T therapy efficacy. **B.** The radiant counts calculation according to bioluminescent imaging results.

Through a succession of bioluminescent imaging for 5 weeks (Figure 7A), the antitumor efficacy of avidin-CAR-T cells was validated. After 2 weeks of slowly increasing (mean values were 5.78, 7.7, and 17.35 radiant counts at before therapy and the 1^st^, 2^nd^ week respectively), the mean value of radiant counts of EGFRvIII^+^ tumors rose to near 100 at the 3^rd^ week post-therapy and then sharply dropped to 36.5 at the 4^th^ week post-therapy, which indicates that the therapy reduced the tumor-burden. In contrast, the radiant counts of EGFRvIII^−^ tumors continuously increased (mean values were 14.77, 16.26, 44.10 and 58.61 at before therapy and the 1^st^, 3^rd^ and 4^th^ week respectively), excluding a slight decline to 7.99 at the 2^nd^ week post-therapy (Figure 7B, Supplemental Figure 2).

## DISCUSSION

Adoptive transfer of T cells with a specific CAR promotes cancer killing and has shown promise for the immunotherapy of human malignancies [25, 26]. Currently, a number of early phase clinical trials are underway that consist of using gene-modified peripheral blood lymphocytes, with CARs directed against a variety of tumor antigens [26-29]. In the current study, we used a recently reported strategy [20, 21] to treat EGFRvIII expressing glioma xenografts. We constructed avidin-CAR lentivirus plasmids and bioengineered T cells to express the CARs. To ensure the functional activity of the avidin-CAR-T cells, we analyzed their targeting, functional activity and cytotoxicity. CAR-T cells with high expression of avidin had phenotypes characteristic of cytotoxic T cells and they secreted significant amounts of IFN-γ. Avidin-CAR-T cells were then directed against the antigen EGFRvIII, as it is often expressed by malignant cancer cells and has been associated with a poor prognosis [7] and has been suggest to also be expressed by cancer stem cells [30]. We biotinylated 4G1 (biotin-4G1) and validated its capability to specifically bind EGFRvIII but not wild-type EGFR *in vitro*. After EGFRvIII on tumor cells was bound with biotin-4G1, avidin-CAR-T cells were either added to the culture for *in vitro* analysis or adoptively transferred into tumor bearing mice for *in vivo* analysis. The *in vitro* and *in vivo* analysis of avidin-CAR-T cell cytotoxicity indicated that the avidin-CAR-T cells were able to target and kill EGFRvIII expressing tumor cells.

Recent efforts to improve the antitumor efficacy of CAR-based therapies focus largely on the improvement of CAR design, including antigen receptor development [25, 28, 31, 32] or the introduction of costimulatory molecules [17, 33]. However, despite significant progress, some major limitations have not been solved and significant challenges still exist for the clinical application of CAR-T cells [34]. For instance, one limitation is the difficulty in visually observing the T cells *in vivo*, which is applicable to both the regular CAR and avidin-CAR strategies.

The avidin-CAR-T cells target TAAs and therefore one of the determining factors for this system's therapeutic efficacy depends on the binding affinity and pharmacokinetics of biotinylated molecules and TAAs. Biotinylated polypeptides or antibodies should have different pharmacokinetics and binding affinities *in vivo* and *in vitro*, so it is necessary to develop a reliable method to evaluate the binding affinity and pharmacokinetics of different biotinylated molecules *in vivo* before and through CAR-T cells therapy process. For this reason, this study tried to use an optical molecular imaging approach to visualize the therapy. Through imaging study, we should achieve two goals: first, determine the specificity of pre-target and target; second, the appropriate time point for T cells adoptive transfer.

For confirming the specific binding of biotin-4G1, we labeled biotin-4G1 with near-infrared dye (biotin-4G1-dye) and injected it into mice bearing EGFRvIII positive or negative tumors. The uptake of biotin-4G1-dye by tumor cells *in vivo* was then analyzed. After biotin-4G1-dye injection, streptavidin-Cy7 was injected into experimental animals to confirm the binding of streptavidin to the target. Results of imaging study validated the high specificity of this biotin-avidin-T cells system.

On the other hand, one of the solutions to achieve more therapeutic benefits is to find an appropriate time point for transferring T cells. At that time point, more transferred T cells target tumor while less target normal tissue in order to reduce unnecessary cytotoxic effect to normal tissue. To determine this crucial time point, we performed optical imaging and bio-distribution study to evaluate accumulation of biotin-4G1-dye in tumors and normal tissues. From the results, we found accumulation of biotin-4G1-dye in tumors reached the peak at 4 and 24 h post-injection but rapidly declined at 48 and 72 h post-injection. So it seems that both 4 and 24 h are reasonable time for T cells transfer. However, in consideration of abundant non-specific uptake of biotin-4G1-dye in normal tissues and relative inferior T/NT values at 4 h, we finally determined 24 h as the optimal time point for T cells adoptive transfer.

In future, we will directly label avidin-CAR-T cells or regular CAR-T cells and biotinylated molecules with appropriate isotopes for PET or SPECT imaging and we expect this strategy to make an important contribution to the clinical application of CAR-T cells. To our knowledge, this is the first study to employ molecular imaging in the CAR therapy research field to provide a real-time approach for evaluating the binding specificities and determining time point for T cells transfer *in vivo*. We strongly recommend widespread application of molecular imaging in future CAR therapy research.

In conclusion, we have confirmed the technical and therapeutic feasibility of the biotin-avidin system in adoptive immunotherapy of EGFRvIII positive gliomas and we developed optical molecular imaging approaches to evaluate it.

## MATERIALS AND METHODS

### Plasmids construction, cell lines and labeling kits

The retroviral plasmid for the expression of anti-EGFRvIII CARs, pMSGV1-huAb139scFv-hCD8.28BBZ, was kindly provided by Prof. Richard Morgan. pRSET-mSA-EGFP was a gift from Sheldon Park (Addgene plasmid #39862) [22]. pITA is a 2nd generation Lentiviral Plasmis. It is derived from pZSG and the promoter is switched from a UBC to an EF1 alpha promoter. pITA can be used for cDNA expresion with puro resistance. For construction of the third generation avidin-CAR lentiviral vector, pITA-avidin-CAR, the DNA fragment of streptavidin was derived by PCR from pRSET-mSA-EGFP. The streptavidin was then subcloned (5′- ccgCTCGAGATGGCTGAAGCTGGTATCACCG and 5′-ataagaatGCGGCCGCTAATGG TGGTGATGGTGATGGG) into the XhoI and NotI site of pMSGV1-huAb139scFv-hCD8.28BBZ to replace the previous plasmid huAb139scFv. The open reading frame (ORF) from streptavidin to CD3-ZETA was amplified by PCR (5′-ATGGCTGAAGCTGGTATCACCG and 5′-TTAGCGAGGGGGCAGGG) and inserted into a pGEM-T Easy Vector and the NotI (5′- TAAACCGTCCGCTGCTTCCaCGGCCGCATT and 5′- tGGAAGCAGCGGACGGTTTAACTTTGGTGAAGG) and BamHI (5′-CTGGTACAACCAGCTGGGtTCCACCTTCATCG and 5′-aCCCAGCTGGTTGTACCAGGTGCCGGTGAT) sites in the ORF were removed by site-directed Mutagenesis. The Streptavidin-hCD8.28BBZ ORF was then subcloned 5′- ataagaat GCGGCCGCATGGCTGAAGCTGGTATCACCG and 5′-cgcGGATCCTTAGCGAGGGGGCAGGG) into the NotI and BamHI site of the lentivector pITA (Figure 1A). For construction of the luciferase lentiviral vector pITA-Luci, the luciferase DNA fragment was derived by PCR and subcloned into the NotI and BamHI site of the lentiviral vector, pITA.

F98_npEGFR_ and F98_npEGFRvIII_ rat glioblastoma cell lines were purchased from the American Type Culture Collection (Rockvill, MD, USA) and were bioengineered to express luciferase. Cells were cultured in high glucose DMEM supplemented with 10% fetal bovine serum and 0.2 mg/ml G-418 at 37°C with 5% CO_2_.

Kits for antibody biotinylation and near-infrared dye labeling were both purchased from Thermo Fisher Scientific Inc. (Waltham, MA, USA) and stored at −20°C.

### Isolation, activation and lentiviral infection of human T cells

All peripheral blood samples were obtained from healthy volunteers under an institutional review board approved protocol. To isolate peripheral blood mononuclear Cells (PBMC), the red blood cells and platelets were removed with PBMC isolation kits according to the manufacturer's instruction (AllCells, Alameda, CA, USA). For T cell separation and activation, the acquired cell pellet was re-suspended in media X-VIVO15 and cultured. After 5 h, the cells were transferred to another flask for culture in media X-VIVO15 supplemented with 50 IU of human recombinant interleukin-2, anti-CD3 and anti-CD28 monoclonal antibodies (Becton-Dickinson, Rutherford, NJ, USA) for T cell activation.

For lentiviral infection, T cells were transferred to a 6-well plate at 5×10^6^ cells/well in 2 ml. After 12–24 h of activation, avidin-CAR lentiviral particles and 8 μg/ml polybrene was added to each well and the cells were incubated for 12–16 hours. Virus titres were adjusted to infect 90–99% of cells. Additionally, 8 μg/ml polybrene without lentiviral particles was added to another well as a control. Next, the medium containing lentiviral particles were removed and 2 ml fresh medium with 50 IU/ml of IL-2 was added to each well.

### Activated T cell phenotype analysis and cytokine secretion assays

The expression of avidin-CAR on T cells was detected by B5F (Biotin-5-fluorescein conjugate, Sigma, St. Louis, MO, USA) or by flow cytometry using mouse-avidin monoclonal antibodies (Santa Cruz, Dallas, TX, USA) as the primary antibody and rabbit anti-mouse IgG conjugated to FITC (Santa Cruz) as the secondary antibody (Becton-Dickinson). The following monoclonal antibodies were used for T cell phenotype determination: mouse anti-Human CD3-CF594, mouse anti-human CD4-FITC, mouse anti-human CD8-PE, mouse anti-human CD25-APC and mouse anti-human CD56-PE-CY7 (Becton-Dickinson). The presence of IL-2, IL-4, IL-6, IL-10, interferon-γ (IFN-γ), tumor necrosis factor (TNF) and IL-17 of activated avidin-CAR-T cells in culture supernatant were analyzed using Human Th1/Th2 Cytokine Kit II (Becton-Dickinson) according to manufacturer's instructions. The cytokines concentration of non-activated avidin-CAR-T cells was also determined as control. Values represent the mean of triplicate wells.

### Biotinylation and dye labeling of the anti-EGFRvIII antibody 4G1

A novel mouse anti-EGFRvIII monoclonal antibody, 4G1, was prepared according to our unpublished study. To prepare biotin-4G1, 1.33×10^−4^ mmol of Sulfo-NHS-Biotin solution (Thermo Fisher) was added to 1 mg of 4G1 dissolved in phosphate-buffered saline (20:1 molar ratio of Sulfo-NHS-Biotin and antibody) and the reaction was incubated at room temperature for 30–60 minutes. The reaction mix was then purified with a Zeba Spin Desalting Column (Thermo Fisher) to remove excess biotin reagent, the flow through of which was the purified biotinylated antibody sample. To measure the level of biotin incorporation, 100 μl of biotin-4G1 was added to a cuvette containing HABA/Avidin. The absorbance was measured at 500 nm and the HABA assay calculation was performed according to the manufacturer's instructions.

To prepare the optical imaging probe, we labeled biotin-4G1 with a near infrared dye, Dylight 680 NHS (Ex/Em: 682/715 nm, Thermo Fisher). Briefly, 140 μg Dylight 680 NHS was added to 2 mg biotin-4G1 solution (molar ratio =10:1) and the reaction was incubated at room temperature for 2 h. The reaction mix was purified with a PD-10 column (Amersham, Piscataway, NJ, USA) to remove excess dye. The collected flow-through solution contained the purified labeled biotin-4G1 (biotin-4G1-dye). The degree of labeling was calculated by analyzing the sample using spectrophotometer at an absorbance of 280nm and 684 nm according to the manufacturer's instructions.

### F98_npEGFR_ and F98_npEGFRvIII_ xenograft model of glioma

All animal experiments were performed in accordance with the Guidelines of Peking University Health Science Center Animal Care and Use Committee. 2×10^6^ tumor cells in 100 μl were injected subcutaneously into the right or both legs of female BALB/c nude mice.

### Evaluation of biotin-4G1's pre-target to EGFRvIII

I*n vitro* tests were performed to verify the binding specificity of biotin-4G1. EGFRvIII expression by F98_npEGFR_ and F98_npEGFRvIII_ cells was analyzed by western blotting and flow cytometry using biotin-4G1 and Dylight 800-rabbit anti-mouse IgG (EarthOx, San Francisco, CA, USA) as the primary and secondary antibodies, respectively. Mouse IgG was used as an isotype primary antibody. For the indirect immunofluorescent assay (IFA), cells grown on LabTek chamber slides were incubated with biotin-4G1 antibodies at 4°C overnight and then incubated with FITC-rabbit anti-mouse IgG at room temperature for 2 h. DAPI was used for nuclear staining. Fluorescence signals were detected using a confocal microscope (TCS SP5; Leica, Germany). For immunohistochemistry (IHC), paraffin sections of F98_npEGFR_ and F98_npEGFRvIII_ xenograft tumors were made. Biotin-4G1 and HRP-rabbit anti-mouse IgG were used as primary and secondary antibodies, respectively. The sections were incubated in diaminobenzidine–hydrogen peroxide solution (Boster, DAB staining kit, Wuhan, China) and examined microscopically.

For *in vivo* molecular imaging, 0.5 nmol of biotin-4G1-dye was intravenously injected into mice when the tumor size reached between 50 and 100 mm^3^. Mice were then anesthetized by inhalation of 2% isoflurane and were injected with 2 mg D-luciferin (SynChem, Inc, IL, USA) dissolved in PBS. Bioluminescent and optical imaging at Ex/Em: 675/720 nm were then performed respectively under an IVIS small animal imaging system (Xenogen, Alameda, CA) at successive time points (4, 24, 48 and 72 h). Besides for biotin-4G1 binding effect evaluation, the optimal time point for *in vivo* avidin-CAR T cell transfer was determined according to imaging results.

### Evaluation of avidin-CAR T cells' re-target

For *in vitro* evaluation of avidin-CAR-T cells binding to biotin-4G1, 20 μg of of biotin-4G1 was added to F98_npEGFRvIII_ cells at a concentration of 1×10^5^ cells in a 6-well plate, which were then incubated at 4°C for 1 h; 20 μg of 4G1 was also added to tumor cells as a control. After the cells were washed three times to remove excess biotin-4G1, 5×10^6^ avidin-CAR T cells were added to each well and the cells were incubated for 1 h. The cells were then washed three times to remove non-binding T cells. Plates were analyzed under a phase contrast microscope to determine avidin-CAR T cells' re-target.

For *in vivo* molecular imaging, 0.5 nmol of biotin-4G1-dye was intravenously injected into mice. 0.5 nmol Strepavidin-Cy7 (Amyjet Scientific Inc. Wuhan, CHN) dissolved in PBS was then intravenously injected into mice at the optimal time, as determined by the pre-target imaging results (mentioned above). After 0.5 h, mice were subjected to bioluminescent imaging after 2 mg of luciferin injection and optical imaging with excitation and emission absorbance of 675/720 nm for Dylight 680 detection and 750/785 nm for Cy7 detection.

### Determination of optimal time for T cells adoptive therapy

Four F98_npEGFRvIII_ tumor-bearing mice were intravenously injected with 10 nmol of biotin-4G-dye and used for optical imaging at 4, 24, 48 and 72 h post-injection. After each time point imaging study, one mouse was sacrificed to bio-distribution analysis. Briefly, mice were killed and dissected, tumors and major organs were collected and wet-weighed. The fluorescence radiant efficiency in these tissues was measured using IVIS imaging system. The results are presented as percentage injected dose per gram of tissue (%ID/g). And the ratios of the levels of tissue %ID/g between the tumors and normal tissues (T/NT ratios) were calculated.

### Therapeutic efficacy evaluation

A modified and simplified cytotoxicity assay was undertaken for *in vitro* evaluation. Briefly, 20 μg of biotin-4G1 was added to F98_npEGFR_ or F98_npEGFRvIII_ cells at density of 1×10^5^ cells in 6-well plates, which were then incubated at room temperature for 1 h. The cells were then washed three times and avidin-CAR T cells (effector cells) were added to the pre-targeted cells at effector-to-target ratios (E:T) of 0:1, 10:1, 20:1 and 50:1. The cells were incubated at room temperature for 4 h and then washed three times. The cells were then trypsinized, suspended in phosphate-buffered saline and stained with propidium iodide for 3 min before flow cytometric analysis. Tumor cells stained with propidium iodide were regarded as non-viable cells.

For *in vivo* antitumor efficacy evaluation, F98_npEGFR_ or F98_npEGFRvIII_ tumor-bearing mice were intravenously injected with 100 ng of biotin-4G when the total radiant efficiency of each tumor reached 20 counts. At the optimal time point post injection of biotin-4G (as determined by *in vivo* 4G1 imaging), 1×10^7^ activated avidin-CAR T cells were intraperitoneally injected. Bioluminescent imaging was performed every week to evaluate the antitumor activity of the avidin-CAR T cells.

### Statistical analysis

Data are expressed as mean ± SD. Statistical evaluation was performed using 2-tailed Student's *t* tests. *P* values less than 0.05 were considered significant.

## SUPPLEMENTARY MATERIAL FIGURES AND TABLES


