# Supplementary material for: Retargeted human avidin-CAR T cells for adoptive immunotherapy of EGFRvIII expressing gliomas and their evaluation via optical imaging
Source: Oncotarget. 2015 Jun 8;6(27):23735–47. doi: 10.18632/oncotarget.4362 (PMC4695148; doi:10.18632/oncotarget.4362)
Supplement: Supplementary file 1 [file oncotarget-06-23735-s001.pdf]

Retargeted human avidin-CAR T cells for adoptive immunotherapy of EGFRvIII expressing gliomas and their evaluation via optical imaging

Supplementary Material

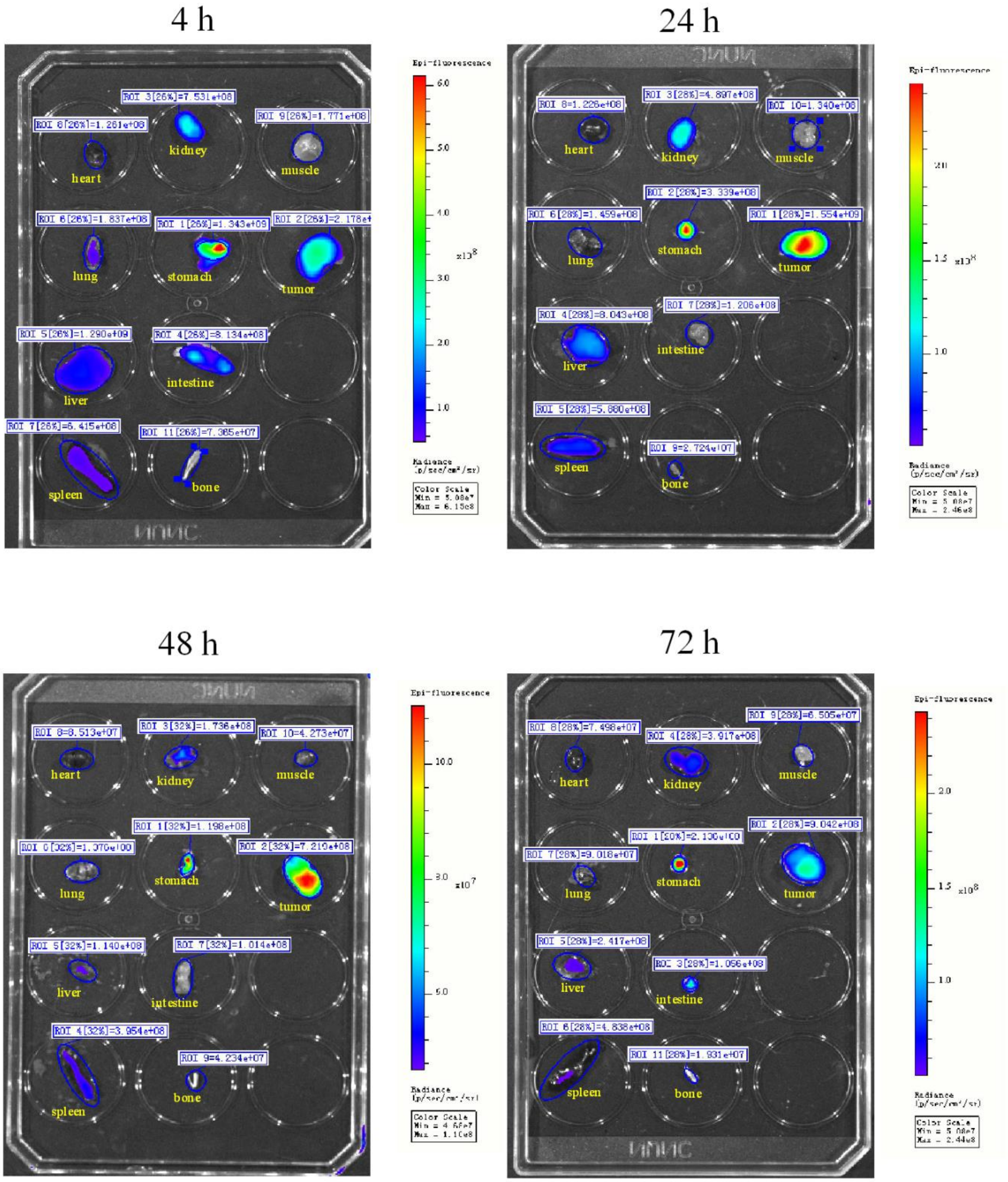

Supplemental Figure 1: Radiant efficiency of isolated tissues The radiant efficiency of isolated tissues containing heart, lung, liver, spleen, kidney, stomach, intestine, bone, muscle and tumor at four time points were analyzed.

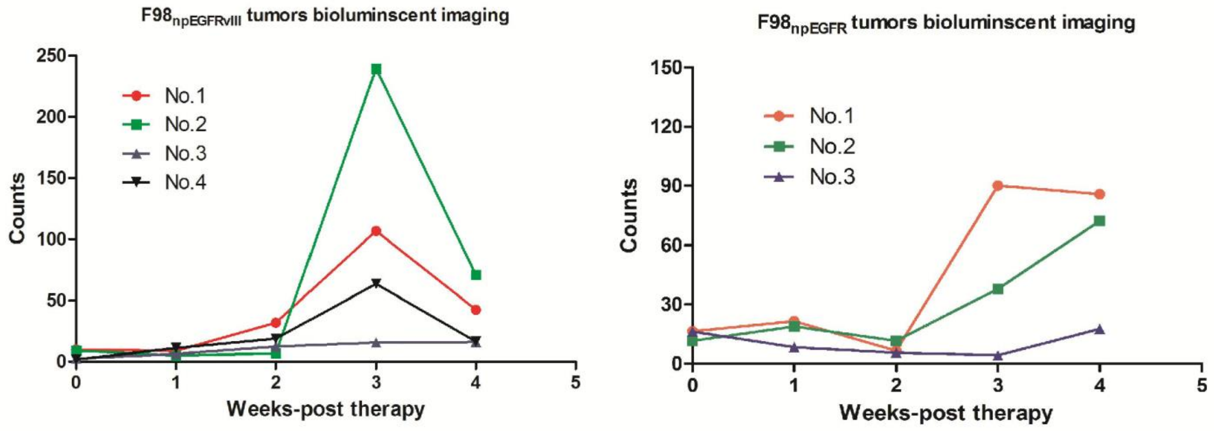

Supplemental Figure 2: Linear chart of bioluminescent imaging. Radiant counts of each numbered mouse through the therapy test were analyzed

Table 1: Cytokine secretion determination (pg/ml)

Sample	IL-2	IFN-γ	IL-4	IL-6	IL-10	TNF	IL-17
**No.1**	15868.7	2961.1	45.3	48.5	21.1	33.7	162.1
**No.2**	6231.3	1327.6	6.9	37.2	7.0	8.4	50.9
**No.3**	12887.9	1045.8	8.1	30.1	6.6	8.7	62.4
**No.4**	11823.8	5579.2	22.6	144.1	21.9	44.9	77.9
**Control**	21120.4	24.4	7.7	4.9	6.8	5.3	43.3

Table 2: Weight of experimental animals' organs at different time points (unit: g)

Organs	4 h	24 h	48 h	72 h
**heart**	0.064	0.063	0.058	0.065
**lung**	0.024	0.024	0.016	0.012
**liver**	0.168	0.225	0.026	0.043
**spleen**	0.09	0.098	0.07	0.098
**kidney**	0.08	0.087	0.051	0.096
**stomach**	0.065	0.061	0.024	0.025
**intestine**	0.065	0.033	0.02	0.011
**bone**	0.006	0.005	0.005	0.004
**muscle**	0.046	0.044	0.015	0.019
**tumor**	0.303	0.247	0.278	0.281

Table 3: Bio-distribution data of biotin-4G1-dye in $F98_{npEGFRvIII}$ glioma bearing xenograft (unit: %ID/g)

Organs	4 h	24 h	48 h	72 h
**heart**	1.83	1.87	1.36	1.07
**lung**	7.12	5.65	8.00	6.99
**liver**	7.14	3.32	4.07	5.23
**spleen**	6.63	5.58	5.25	4.59
**kidney**	8.75	5.23	3.16	3.80
**stomach**	19.21	5.09	4.64	7.94
**intestine**	11.63	3.40	4.71	8.93
**bone**	11.41	5.07	7.87	4.49
**muscle**	3.58	2.83	2.65	3.18
**tumor**	6.68	5.85	2.41	2.99

Table 4: Ratio of tumor/normal tissue (T/NT) in $F98_{npEGFRvIII}$ glioma bearing xenograft

Organs	4 h	24 h	48 h	72 h
**heart**	3.98	3.13	1.77	2.79
**lung**	1.02	1.03	0.30	0.43
**liver**	1.02	1.76	0.59	0.57
**spleen**	1.10	1.05	0.46	0.65
**kidney**	0.83	1.12	0.76	0.79
**stomach**	0.38	1.15	0.52	0.38
**intestine**	0.63	1.72	0.51	0.34
**bone**	0.64	1.15	0.31	0.67
**muscle**	2.03	2.07	0.91	0.94
